# Divergent transmission dynamics and drug resistance evolution of HIV-1 CRF01_AE and CRF07_BC in Tianjin, China (2013–2022)

**DOI:** 10.1186/s12985-025-02704-y

**Published:** 2025-05-08

**Authors:** Zheng Minna, Zhao Hehe, Ning Tielin, Zhao Fangning, Gong Hui, Lyu Fan, Yu Maohe

**Affiliations:** 1 Department of AIDS/STD Control and Prevention, Tianjin Centers for Disease Control and Prevention, Tianjin, 300011 China; 2 Tianjin Key Laboratory of Pathogenic Microbiology of Infectious Disease, Tianjin Centers for Disease Control and Prevention, Tianjin, 300011 China; 3 National Center for AIDS/STD Control and Prevention, Chinese Center for Disease Control and Prevention, Beijing, 102206 China; 4 National Key Laboratory of Intelligent Tracking and Forecasting for Infectious Disease, Beijing, 102206 China

**Keywords:** HIV-1, Phylodynamic analysis, Transmission dynamics, DRMs

## Abstract

**Background:**

Tianjin, a major hub in northern China, faces rising HIV-1 infections dominated by CRF01_AE and CRF07_BC. This study elucidated their divergent transmission patterns and drug resistance dynamics to guide targeted interventions.

**Methods:**

This study included samples identified as CRF01_AE and CRF07_BC subtypes through various methods between 2013 and 2022. BEAST software was used to examine the spatiotemporal transmission patterns of these subtypes in Tianjin. By integrating HIV-TRACE, we constructed high-risk transmission clusters and identified drug resistance mutations (DRMs) based on the Stanford HIV Drug Resistance Database. Finally, the birth–death skyline serial (BDSKY) model was employed to dynamically assess the effective reproductive number (Re) of both subtypes to predict future transmission dynamics.

**Results:**

CRF01_AE might be introduced in 1988 from Henan and Zhejiang, forming multiple small clusters (< 10 nodes) and spreading through both heterosexual and men who have sex with men (MSM) in Tianjin, while CRF07_BC from Chongqing and Guizhou, et al. in 2004, experiencing explosive local transmission and forming a large cluster of 170 nodes primarily among MSM under 30 years old (*P* < 0.05). Phylogenetic analysis indicated that CRF01_AE has a significantly higher evolutionary rate (2.08 × 10⁻^3^ vs. 1.48 × 10⁻^3^ substitutions/site/year, *P* < 0.05), while CRF07_BC demonstrates a greater cluster formation capacity (56.6% vs. 37.1%, *P* < 0.05). CRF01_AE showed a higher mutation occurrence rate (5.18% vs. 2.49%, P < 0.05), particularly with non-nucleoside reverse transcriptase inhibitor (NNRTI) associated mutations (e.g., K101E). Although CRF07_BC had a lower resistance burden, the emergence of K103E mutations suggests a need for vigilance regarding potential decreases in sensitivity to newer NNRTIs. BDSKY modeling revealed that the Re for CRF01_AE dropped below 1 after 2016, whereas CRF07_BC’s Re remains above 1, indicating that the risk of transmission still exists.

**Conclusion:**

Subtype-specific strategies are critical: intensified resistance monitoring for CRF01_AE and cluster-focused interventions for CRF07_BC, particularly among young MSM.

**Supplementary Information:**

The online version contains supplementary material available at 10.1186/s12985-025-02704-y.

## Introduction

HIV-1 genetic diversity in China is characterized by a predominance of circulating recombinant forms (CRFs), accounting for over 95% of infections [1]. Among these, CRF01_AE and CRF07_BC have emerged as the two most prevalent subtypes, driving distinct transmission networks [2]. From 2020 to 2022, these two subtypes accounted for 29.4% and 40.8% of ART-naïve HIV-infected individuals, respectively [3]. CRF01_AE, originating from Southeast Asia, initially spread via heterosexual contact and commercial routes [4], while CRF07_BC, a B/C recombinant, proliferated among injection drug users (IDUs) in southwestern China [5]. Recent national surveillance indicates their increasing dominance in sexual transmission networks, particularly among men who have sex with men (MSM) [6]. These subtypes exhibit divergent epidemiological trajectories. CRF01_AE is associated with higher rates of transmitted surveillance drug resistance mutations (SDRMs), particularly to non-nucleoside reverse transcriptase inhibitors (NNRTIs), complicating first-line antiretroviral therapy (ART) [7]. In contrast, CRF07_BC demonstrates a propensity for forming large molecular clusters, reflecting concentrated transmission within high-risk networks [8]. Such differences underscore the need for subtype-tailored surveillance and intervention strategies.

Tianjin, a port city with 13 million residents, serves as a critical sentinel for HIV-1 transmission dynamics in northern China. Between 2020 and 2022, 98.06% of new cases in Tianjin were sexually transmitted, with MSM accounting for 79.40% [9]. Since 2014, over 80% of untreated HIV-1 infections in Tianjin have been linked to CRF01_AE and CRF07_BC, indicating ongoing local transmission. Despite the dominance of CRF01_AE and CRF07_BC, their phylogeographic origins, transmission clusters, and resistance patterns remain poorly characterized in this region. This study aims to (1) reconstruct the spatiotemporal spread of these subtypes, (2) compare their drug resistance mutations, and (3) model transmission dynamics to inform targeted control measures.

## Methods

### Sources of CRF01_AE and CRF08_BC sequences

Between 2013 and 2022, we collected 1713 plasma samples from newly diagnosed, ART-naïve HIV-1 positive patients. Demographic information of the study subjects was recorded through face-to-face interviews prior to plasma sample collection. The sample collection was part of routine HIV/AIDS surveillance activities in Tianjin, which complied with the regulations and ethical requirements of the national health authorities. Viral RNA was extracted from 140 $\mu$ L of plasma using a QIAamp Viral RNA Mini Kit (Qiagen, Valencia, CA, USA). The RNA then underwent reverse transcription PCR and nested PCR to amplify a partial HIV-1 *pol* coding region (HXB2: 2253–3306), covering the entire protease and the first 300 codons of the reverse transcription genome. PCR products were purified using a QIAquick PCR Purification Kit (Qiagen, Germany) and sent to Tianjin Kingmed Diagnostics Group Co. for sequencing. Sequences were trimmed and assembled with Sequencher v5.4.5(GeneCodes, Michigan, USA), aligned using ClustalW tool in MEGA v7.0(Sudhir Kumar, Arizona State University). We first used COMET v2.2 [10] to identify HIV-1 subtypes. For sequences that could not be classified by COMET v2.2, we then used phylogenetic analysis as well as the RIP and HIV-1 blast tools from the Los Alamos National Laboratory (LANL) to determine their subtypes. The reference sequences for phylogenetic analysis also came from LANL, covering the major HIV-1 subtypes. Finally, we selected sequences identified as CRF01_AE and CRF07_BC for further analysis.

### Datasets construction of CRF01_AE and CRF07_BC in this study

In addition to the 1,199 sequences (676 CRF01_AE sequences and 523 CRF07_BC sequences) selected in this study, we retrieved all CRF01_AE (4,344) and CRF08_BC (657) sequences from the LANL database. In principle, the selection of representative strains should cover all years and regions in the dataset and each branch of the phylogenetic tree. Of these sequences, questionable and low-quality sequences were eliminated, including those with 5% ambiguous bases, clones, laboratory-adapted strains, and misnamed sequences that belonged to other countries or subtypes. Using MEGA v7.0, we constructed a neighbor-joining tree to select representative strains. Based on the geographical and temporal representation of each sequence, we randomly selected 310 CRF01_AE and 246 CRF07_BC representative strains from each branch of the tree to describe the evolutionary characteristics of the two subtypes in Tianjin on a national scale.

### Evolutionary characteristics and phylogeographic inference of CRF01_AE and CRF07_BC

To elucidate the transmission dynamics and spatiotemporal migration patterns of CRF01_AE and CRF07_BC in Tianjin Province, we conducted a comprehensive phylodynamic analysis using 310 CRF01_AE and 246 CRF07_BC partial *pol* sequences processed with BEAST v 1.10.4 [11]. A root-to-tip regression analysis was conducted to examine the equal distribution of temporal signals in the dataset using TempEst v1.5.3 [12]. Furthermore, Bayesian phylodynamic inferences were used to investigate the evolutionary characteristics of subtype CRF01_AE and CRF07_BC using the Markov Chain Monte Carlo (MCMC) method implemented in BEAST software v1.10.4. The MCMC chains were run for enough iterations to achieve stationarity and adequate mixing for all parameter estimates. The asymmetric substitution model with the BSSVS option in BEAST was used to estimate the diffusion rates between different areas of China, enabling Bayesian factor (BF) calculations to determine the significance of these diffusion rates. The output generated by BEAST was then analyzed in the TRACER program v1.7 [13] to confirm convergence (ESS $>$ 200). TreeAnnotator was used to construct a maximum clade credibility (MCC) tree and then the tree was visualized using iTOLv6.8.1 [14]. For the analysis and visualization of pathogen phylodynamic reconstructions, we employed the SpreaD3 package v0.9.7 [15] to identify significant migration pathways.

### Drug resistance and clustering analysis

HIV-1 SDRMs were identified using the Calibrated Population Resistance (CPR) tool in the Stanford HIV-1 database (https://hivdb.stanford.edu/cpr/form/PRRT/), following the WHO’s definitions for SDRM definitions. HIV-TRACE v0.4.4(www.hivtrace.org) [16] was used to construct molecular transmission networks, and pairwise genetic distance between pairs of sequences were calculated using Hyphy v2.2.4 [17], and a genetic distance threshold of 1% (the genetic distance was calculated based on the Tamura-Nei 93 (TN93) model, and an appropriate threshold for constructing the molecular transmission network was determined by the number of identifiable transmission clusters at different genetic distances)was applied to define molecular transmission clusters. To minimize potential bias due to convergent evolution, codons associated with HIV-1 drug resistance were marked, based on a commonly used subtype B-optimized list [18].

### Estimation of effective reproductive number

The birth–death skyline serial (BDSKY) model in BEAST v2.5 [19] was used to infer changes in effective reproductive number (Re) for CRF01_AE and CRF07-BC over time, providing an estimate of the average number of new infections caused by an infected individual at a specific time during the outbreak. The Re is calculated as the median ratio of the birth and death rates and was estimated for the two subtypes over 3 equidistant time dimensions. The 95% highest probability density(95%HPD) intervals for Re were defined as the smallest intervals containing 95% of the posterior probability of the Re estimate [20]. To ensure the robustness of our analysis, we employed a GTR substitution model with gamma-distributed rate variation and a proportion of invariant sites, along with an uncorrelated log-normal relaxed molecular clock model. The BDSKY model priors were set for becoming Uninfectious Rate (Lognormal [M = 0;S = 1.25;dimension = 10]), origin (Lognormal [M = 3;S = 1]), reproductive Number (Lognormal [M = 0;S = 1.25;dimension = 10]), sampling proportion (Beta [Alpha = 10;Beta = 10]) according the technical guide for HIV transmission networks monitoring intervention [21], and a fixed substitution rate of 3 $\times$ 10^−3^ nucleotide substitutions per site per year [22, 23].The Re estimates were visualized using R software 4.3.3.

### Statistical analysis

Categorical variables were reported as counts and percentages. Continuous variables were analyzed using the Mann–Whitney U-test for nonparametric statistics. Categorical comparisons utilized Chi-square tests, with Fisher’s exact test applied when cell counts were under 5. All analyses were conducted with SPSS v24.0 (Inc., Chicago, IL, USA), and statistical significance was set at 5%.

## Results

### Comparison of demographic

A total of 1,199 HIV-infected individuals were selected to conduct a comparative analysis in this study, including 676 cases (56.38%) with the CRF01_AE subtype and 523 cases (43.62%) with the CRF07_BC subtype. Results showed statistically significant differences between the two subtypes in terms of age distribution, transmission route, initial CD4 count, SDRM incidence, and viral clustering (*P* < 0.05). Specifically, the proportion of individuals under 30 years in the CRF07_BC group (41.49%) was higher than the 36.39% observed in the CRF01_AE group. MSM was the primary transmission route, accounting for 96.89% and 97.51% in the two groups respectively. The proportion of patients with initial CD4 counts below 350 × 10^9^/L was 60.50% in the CRF01_AE group, higher than the 50.48% in the CRF07_BC group, and the SDRM incidence was 5.18% in the CRF01_AE group, higher than the 2.49% in the CRF07_BC group. The CRF07_BC subtype was more prone to form large transmission clusters (39.80%), whereas the CRF01_AE subtype had a higher proportion of sporadic cases (62.10%) (Table 1).

**Table 1:** Description of the demographic characteristics of HIV-infected individuals in Tianjin from 2013 to 2022

	Total No. (%)	CRF01_AE (%) No. (%)	CRF07_BC No. (%)	χ2
Total	1199 (100)	676 (100)	523 (100)	
*Age*				7.846*
< 30	463 (38.62)	246 (36.39)	217 (41.49)	
30 ~	451 (37.61)	251 (37.13)	200 (38.24)	
45 ~	222 (18.52)	136 (20.12)	86 (16.44)	
60 ~	63 (5.25)	43 (6.36)	20 (3.83)	
*Gender*				3.105
Female	4 (0.33)	4 (0.59)	0 (0)	
Male	1195 (99.67)	672 (99.41)	523 (100)	
*Transient population*				1.375
No	715 (59.63)	413 (61.09)	302 (57.74)	
Yes	484 (40.37)	263 (38.91)	221 (42.26)	
*Marital status*				5.465
Single	740 (61.72)	409 (60.50)	331 (63.29)	
Married	206 (17.18)	131 (19.38)	75 (14.34)	
Divorced/widowed	253 (21.1)	136 (20.12)	117 (22.37)	
*Risk group*				6.721^*^
HST	28 (2.34)	20 (2.96)	8 (1.53)	
MSM	1165 (97.16)	655 (96.89)	510 (97.51)	
MSM + HST	3 (0.25)	0 (0)	3 (0.57)	
IDU	3 (0.25)	1 (0.15)	2 (0.38)	
*Education*				0.278
Junior middle school and below	347 (28.94)	196 (28.99)	151 (28.87)	
High or technical school	313 (26.11)	180 (26.63)	133 (25.43)	
Junior college or above	539 (44.95)	300 (44.38)	239 (45.7)	
*STDs*				0.021
With	264 (22.02)	149 (22.04)	115 (21.99)	
Without	902 (75.23)	508 (75.15)	394 (75.33)	
Unknown	33 (2.75)	19 (2.81)	14 (2.68)	
*First CD4 count (10*^*9*^*/L)*				12.035^*^
< 350	673 (56.13)	409 (60.50)	264 (50.48)	
> = 350	526 (43.87)	267 (39.50)	259 (49.52)	
*SDRMs*				5.560^*^
With	48 (4.00)	35 (5.18)	13 (2.49)	
Without	1151 (96.00)	641 (94.82)	510 (97.51)	
*Clustering*				109.418^*^
In large clusters	299 (24.90)	91 (13.50)	208 (39.80)	
In small clusters	253 (21.10)	165 (24.40)	88 (16.80)	
Singleton	647 (54.00)	420 (62.10)	227 (43.40)	

HST: Heterosexual transmission; MSM: Men who have sex with men; IDU: Injection drug users. ^*^P values < 0.05 were considered statistically significant by Chi-square test, Fisher’s exact test was used when the number of cells was less than 5

### Evolutionary characteristics and phylogeographic inference of CRF01_AE and CRF07_BC in Tianjin

**Fig. 1:**
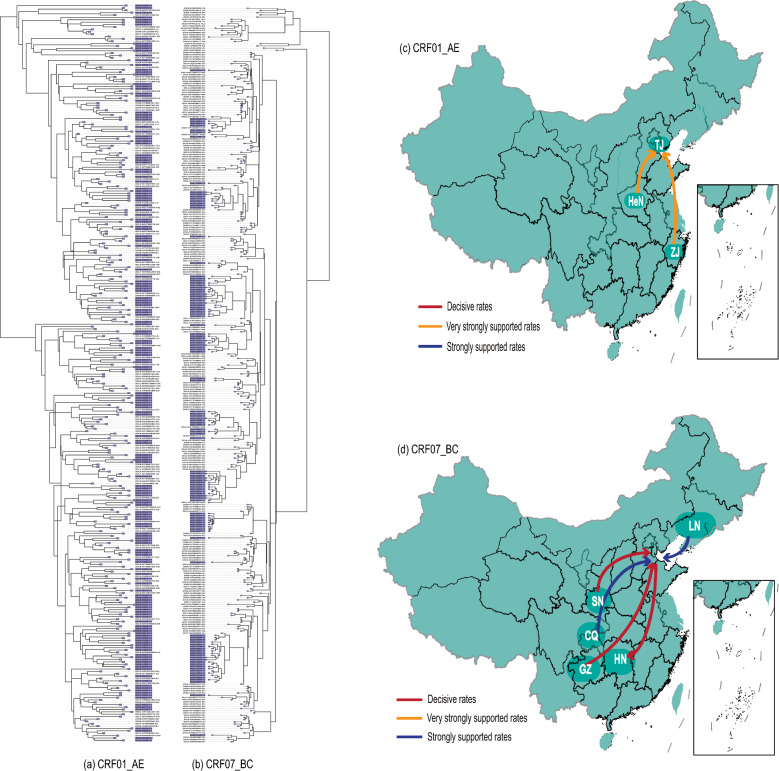
**a**, **b** MCC trees and map of geographic location transition based on CRF01_AE and CRF07_BC sequences in this study. Purple areas indicate Tianjin strains in this study. **c**, **d** Red arrows indicate decisive rates with BF ≥ 1000; orange arrows indicate very strongly supported rate with 100 ≤ BF < 1000; blue arrows indicate strongly supported rates with10 ≤ BF < 100

Phylogenetic analysis showed that the evolutionary rate of CRF01_AE is 2.08 $\times$ 10^−3^ subs./site/year (95%HPD:1.79–2.38 $\times$ 10^−3^ subs./site/year), while the CRF07_BC is 1.48 $\times$ 10^−3^ subs./site/year (95%HPD:1.24–1.72 $\times$ 10^−3^ subs./site/year). Temporal inference analysis revealed that CRF01_AE was introduced into Tianjin around 1988 (95% HPD: 1984–1992), whereas CRF07_BC was introduced approximately in 2004 (95% HPD: 2003–2005) (Table 2). To investigate the origin and national transmission patterns of CRF01_AE and CRF07_BC, spatial transmission dynamics were inferred based on discrete geographic data. MCC trees showed that CRF01_AE primarily divided into two evolutionary branches, with strains distributed more diffusely across the phylogenetic tree, indicating higher genetic diversity. In contrast, CRF07_BC strains clustered predominantly in a single independent evolutionary branch, suggesting lower genetic diversity (Fig. 1). Bayesian phylogeographic analysis suggested that the CRF01_AE strains circulating in Tianjin likely originated from cross-regional transmission from Henan and Zhejiang provinces (Bayesian posterior probability > 0.95). Notably, the Bayesian diffusion model indicated that the most probable transmission route for CRF07_BC was from Shaanxi and Guizhou provinces into Tianjin (BF > 1000). Additionally, a geographic transmission analysis based on Markov transition probabilities revealed a significant trend of CRF07_BC diffusion towards Hunan province, further elucidating the spatial spread of this subtype.

**Table 2:** Evolutionary rate and tMRCA of CRF01_AE and CRF07_BC

Evolutionary Rate ($\times$ 10^−3^ subs./site/year) (95%HPD)		tMRCA(95%HPD)	
CRF01_AE	CRF07_BC	CRF01_AE	CRF07_BC
2.08(1.79–2.38)	1.48(1.24–1.72)	1988(1984–1992)	2004(2003–2005)

### Analysis of clustering characteristics

**Fig. 2:**
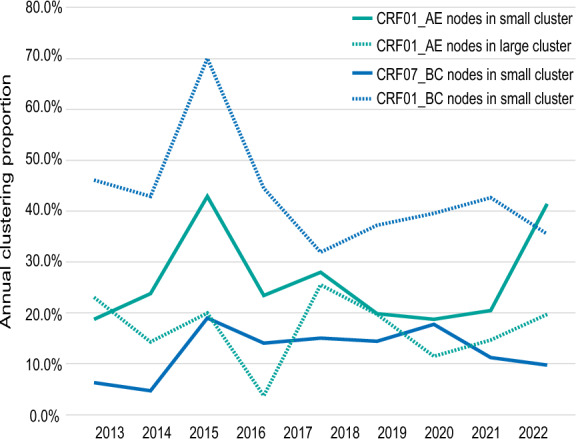
Annual clustering proportion of CRF01_AE and CRF07_BC

We employed molecular transmission network analysis to investigate the differential transmission dynamics between CRF01_AE and CRF07_BC in Tianjin. Network reconstruction revealed distinct clustering patterns: 37.13% (256/676) of CRF01_AE sequences formed 84 transmission clusters (size range: 2–12), whereas CRF07_BC demonstrated significantly higher clustering capacity with 56.60% (296/523) of sequences coalescing into 46 clusters (size range: 2–170; χ^2^ = 109.148, *P* < 0.05). Notably, CRF07_BC exhibited a pronounced propensity for large cluster formation (≥ 5 nodes), suggesting enhanced transmission potential compared to CRF01_AE (Table 1). Temporal analysis identified a progressive decline in overall clustering rates and large cluster proportions for both subtypes since 2016, indicative of reduced transmission events following epidemic control measures. However, small cluster dynamics (2–4 nodes) displayed non-linear temporal patterns, decreasing from 2016 to 2020 followed by a resurgence in subsequent years (Fig. 2). This biphasic pattern may reflect evolving transmission networks or modifications in public health surveillance strategies. Multivariate analysis identified two critical factors associated with subtype-specific transmission differences (Table S1). First, SDRMs showed distinct prevalence patterns between subtypes, potentially influencing transmission fitness. Second, age stratification revealed significantly higher proportions of young cluster participants (< 30 years) in CRF07_BC networks compared to CRF01_AE (45.95% vs. 37.89%; χ^2^ = 8.228, *P* = 0.039), suggesting differential age-specific transmission risks. This epidemiological pattern implies that CRF07_BC transmission networks may be more strongly associated with youth-dominated risk behaviors or social networks.

### Drug resistance characteristics

**Fig. 3:**
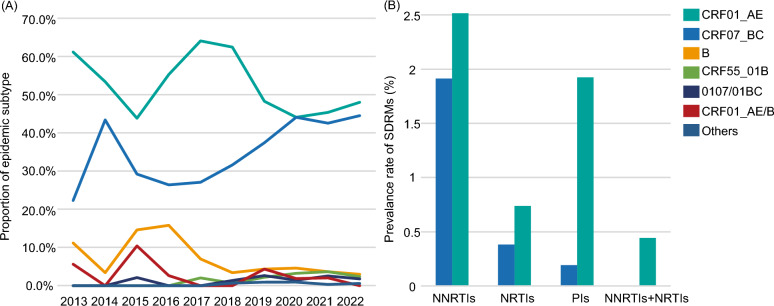
The annual variation trend of subtypes and prevalence of SDRMs

**Fig. 4:**
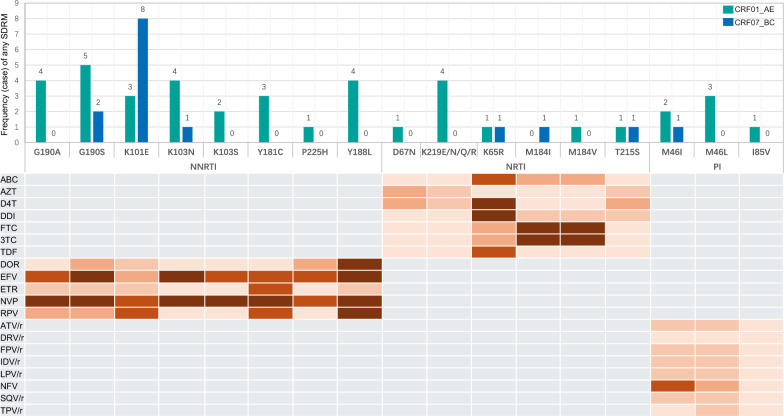
Frequency (case) and characteristic of SDRMs. The color depth of the orange blocks represents different levels of drug resistance. The lightest block indicates “susceptible,” and the levels of resistance increase as follows: Potential Low-level Resistance, Low-Level Resistance, Intermediate Resistance, and High-Level Resistance

A systematic analysis was performed on the prevalence and patterns of SDRMs in HIV-1 CRF01_AE and CRF07_BC in Tianjin. Statistical results showed that the overall SDRM prevalence in CRF01_AE was significantly higher than in CRF07_BC (5.18% vs. 2.49%), with the greatest discrepancy observed in protease inhibitor-associated SDRMs (1.92% vs. 0.19%). By drug class, the prevalence of nucleoside reverse transcriptase inhibitor (NRTI), NNRTI, and combined NRTI + NNRTI mutations in CRF01_AE was 0.74%, 2.07%, and 0.45% respectively, compared to 0.39%, 1.91%, and 0.00% for CRF07_BC (Table 1 and Fig. 3). Analysis of specific mutation patterns revealed notable subtype-specific resistance loci in CRF01_AE, particularly NRTI-SDRMs dominated by K219R/Q/E (0.59%), a characteristic absent in CRF07_BC. In NNRTI-SDRMs, CRF07_BC was most frequently associated with the K103S/E mutation (1.15%), which maintains sensitivity to Rilpivirine (RPV) but confers high-level resistance to Efavirenz (EFV) and Nevirapine (NVP). In contrast, CRF01_AE was highly enriched with the K101E mutation (0.74%), which leads to a graded reduction in susceptibility to EFV, RPV, and NVP. Of note, a marked heterogeneity in PI-SDRMs was observed between the two subtypes: CRF01_AE frequently harbored M46I/L alone or in combination with I86V, which can confer low-to-moderate resistance to Nelfinavir (NFV) and potential low-level resistance to Lopinavir/ritonavir (LPV/r) (0.74% vs. 0.19%). This difference is visually presented in Fig. 4. Further molecular evolution analysis based on a phylogenetic dynamics model indicated that the proportion of strains carrying SDRMs was significantly higher in CRF01_AE lineages than in CRF07_BC (6.64% vs. 2.03%), suggesting that CRF01_AE may possess greater transmission efficiency for drug-resistant variants.

### Modelling effective reproductive number

**Fig. 5:**
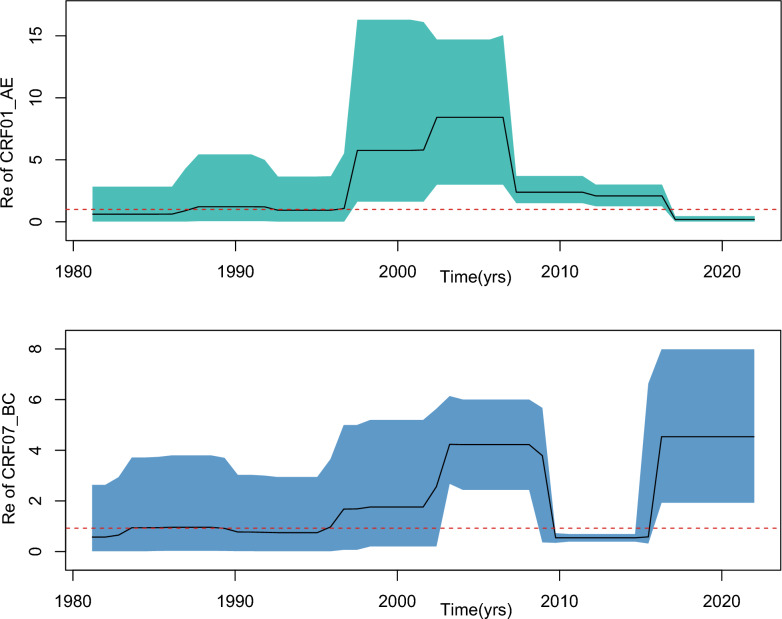
Median of the Re of different subtypes and their HPD intervals. The red line represents the reference value 1

This study used the BDSKY model to analyze the transmission dynamics of HIV-1 CRF01_AE and CRF07_BC subtypes in Tianjin. The results revealed significant heterogeneity in their time-based changes in the Re (Fig. 5). Between 1990 and 2010, Re for CRF01_AE showed exponential growth, peaking at 8 (95% HPD: 3–17.1) from 2004 to 2008, with a growth rate considerably higher than that of CRF07_BC. In contrast, CRF07_BC maintained a stable Re range of 1.8–3.5 (95% HPD: 1.2–4.0) during its initial introduction (1998–2002), followed by a slow cumulative increase that reached 4 (95% HPD: 2–7.8) in 2018. In 2016, however, their trajectories reversed: CRF07_BC became the dominant circulating strain, while CRF01_AE began to decline, indicating the latter had achieved a regional transmission equilibrium. The parameter differences were statistically supported by MCMC convergence diagnostics (ESS > 200) and Bayesian factor analyses.

## Discussion

This investigation delineates distinct evolutionary trajectories and transmission dynamics between CRF01_AE and CRF07_BC HIV-1 subtypes in Tianjin, China, with critical implications for regional epidemic control. CRF01_AE entered the population earlier (in 1988), accumulating more DRMs under prolonged selective pressure and forming multiple cross-provincial transmission chains. In contrast, CRF07_BC primarily spreads through younger MSM, showing a distinct clustering effect that highlights uneven distribution of drug resistance and transmission risk among different subtypes. By comparing changes in the Re and the characteristics of TCs between these two subtypes, public health authorities can optimize intervention strategies and develop more targeted HIV prevention and control measures suited to different populations and regions.

The CRF01_AE subtype, which has been circulating in Tianjin since 1988, showed a higher evolutionary rate (2.08 × 10^−3^ subs/site/year) compared to CRF07_BC (1.48 × 10⁻^3^ subs/site/year), which was introduced later in 2004. Spatiotemporal reconstruction identified fundamentally distinct dissemination patterns. CRF01_AE established early multi-provincial linkages (Zhejiang, Henan) and formed diffuse small clusters (< 10 nodes) across diverse risk groups (heterosexual/MSM), complicating targeted interventions. Conversely, CRF07_BC propagated through explosive local transmission post-2004, generating a massive 170-node cluster concentrated among young MSM (< 30 years). The subtype’s geographical confinement and tight network structure likely reflect behavioral synergies between high-risk MSM subpopulations and viral adaptation to host immune landscapes [24]. CRF01_AE, due to its early introduction and rapid spread, has had a significant public health impact across multiple regions. In contrast, CRF07_BC has developed more slowly and remains geographically confined, possibly leading to larger but more localized transmission clusters.

Investigation of transmission clusters can identify key characteristics of the underlying risk network to guide intervention efforts to improve outcomes and prevent additional infections. In this study, the overall clustering proportion of both CRF01_AE and CRF07_BC has shown a downward trend in Tianjin since 2016. Its complex transmission network, involving both heterosexual and homosexual routes, complicates control efforts with smaller and scattered clusters. Conversely, CRF07_BC is more prevalent among those under 30, forming large clusters, including one with 170 individuals, highlighting strong transmission within China’s MSM population. This spread is linked to high-risk behavior, low prevention awareness, social stigma, and limited interventions, prompting the need for targeted measures in high-risk groups [25]. Additionally, this trend also aligns with the phenomenon that there was a notable decline in the proportion of CRF01_AE cases from 37.3% in 2004–2007 to 29.4% in 2020–2022, while CRF07_BC cases increased significantly from 24.1 to 40.8% in China [26]. Subtype differences may stem from viral traits and host factors. CRF01_AE might accelerate disease progression via higher replication rates or immune escape, underscoring early detection and intervention to curb rapid disease and drug-resistant virus spread. CRF07_BC’s larger clusters suggest concentrated transmission in high-risk groups, matching findings from other cities [27–29].

The relationship between SDRMs and HIV-1 molecular transmission networks is essential for understanding the dynamics of HIV-1 epidemics and guiding effective public health strategies. In this study, we compared the drug resistance profiles and transmission characteristics of the CRF01_AE and CRF07_BC subtypes in Tianjin, revealing significant differences in their prevalence and clustering of SDRMs. This accelerated evolution of CRF01_AE is likely driven by prolonged exposure to NNRTI-based first-line ART regimens, resulting in a higher accumulation of drug resistance mutations, particularly K101E, a non-polymorphic mutation associated with broad resistance to NNRTIs such as EFV and NVP. The presence of K101E, when coexisting with other mutations, further compromises drug sensitivity and raises concerns about the efficacy of standard NNRTI-based regimens. In contrast, CRF07_BC exhibited a lower prevalence of resistance mutations, particularly to NNRTIs, with the most common mutation being K103E, which confers resistance to NVP and EFV but maintains sensitivity to newer NNRTIs like RPV. The lower prevalence of SDRMs in CRF07_BC compared to CRF01_AE may be attributed to both its later introduction (2004 vs. 1988) and shorter cumulative exposure to ART pressures. Additionally, the gradual adoption of improved treatment paradigms during CRF07_BC’s epidemic phase likely played a role. For instance, integrase strand transfer inhibitor (INSTI)-based regimens, recommended by the WHO since 2019 [30] and increasingly implemented in China after 2020 [31], exhibit a higher genetic barrier to resistance compared to NNRTI-based therapies. As CRF07_BC became dominant in Tianjin during the late 2010s, the broader availability of INSTIs may have reduced selective pressure for NNRTI-associated mutations (e.g., K103E) in treatment-naïve populations. This aligns with national trends showing declining NNRTI resistance rates following INSTI scale-up [32]. Nevertheless, CRF07_BC’s shorter ART exposure period remains a primary factor, as resistance development requires prolonged selective pressure. Furthermore, the prevalence of PI-related mutations, such as M46I/L, was more common in CRF01_AE, leading to low to moderate resistance to PI-based regimens. The higher prevalence of SDRMs in CRF01_AE, especially those associated with NNRTIs, underscores the critical need for continuous monitoring of drug resistance in HIV-1 subtypes, particularly in regions where NNRTI-based regimens are widely used. These findings highlight the complex interplay between viral evolution, ART pressure, and the emergence of drug resistance, emphasizing the importance of adapting ART strategies to the evolving molecular epidemiology of HIV. The widespread implementation of INSTI-based regimens in China is highly feasible and increasingly prioritized. Since 2019, China’s National Free Antiretroviral Treatment Program has incorporated dolutegravir (DTG) and bictegravir (BIC) as first-line therapies, supported by centralized procurement policies that significantly reduced drug costs and improved accessibility [26]. By 2022, INSTIs accounted for over 60% of newly initiated ART regimens in major urban centers like Beijing [33] and Shanghai [34], reflecting rapid clinical adoption. This transition is particularly relevant for CRF07_BC-dominated epidemics, as INSTIs’ high genetic barrier to resistance could suppress the emergence of NNRTI-associated mutations (e.g., K103E) in treatment-naïve populations. However, challenges persist in rural and resource-limited regions, where NNRTI-based regimens remain prevalent due to infrastructural and financial constraints [35]. Strengthening INSTI accessibility in these areas, coupled with targeted education for healthcare providers, will be critical to fully realizing their potential in curbing HIV transmission and resistance evolution. Nationally, the declining prevalence of NNRTI-associated SDRMs since 2020 underscores the success of INSTI scale-up [32], further justifying their role as a cornerstone of China’s HIV control strategy.

BDSKY analysis has revealed critical turning points in the transmission dynamics of CRF01_AE and CRF07_BC in Tianjin. For CRF01_AE, its Re has dropped below 1 since 2016, aligning with the nationwide decline in prevalence due to expanded ART coverage and the “treat when found” policy [36]. This indicates that strengthened control measures and rising public awareness have effectively curbed CRF01_AE transmission. In contrast, CRF07_BC has experienced a resurgence since 2016, with its Re consistently exceeding 1, suggesting ongoing spread despite current prevention efforts. This resurgence may be tied to the stronger transmissibility of CRF07_BC within densely populated MSM networks and among younger individuals with higher risk tolerance—factors that highlight significant gaps in targeted interventions [34, 37]. The differences in transmission dynamics between these two subtypes underscore their distinct epidemiological characteristics. CRF01_AE’s broader geographic distribution and higher resistance burden pose a potential threat for cross-regional transmission of drug-resistant mutations [38], whereas the localized spread of CRF07_BC requires more precise strategies to disrupt specific transmission clusters [39]. CRF01_AE demonstrates more explosive growth, while CRF07_BC exhibits a slower but steady upward trend in Re, signaling those factors like population susceptibility, transmission routes, and control measures greatly influence transmission dynamics [40]. Although CRF07_BC’s overall growth is slower, its continued rise in Re indicates an urgent need for enhanced monitoring and targeted interventions, particularly among high-risk populations [9]. Overall, these findings highlight the necessity of adopting differentiated public health strategies for distinct HIV-1 subtypes—both to prevent a resurgence of CRF01_AE and to strengthen surveillance and control efforts for CRF07_BC, especially within high-risk groups and transmission networks.

Furthermore, CRF01_AE’s broader geographical dissemination and resistance burden position it as a persistent threat for multi-regional SDRM spread, whereas CRF07_BC’s localized dominance necessitates hyper-focused cluster disruption. suggest that CRF01_AE exhibits stronger explosive transmission potential after entering the host population, possibly attributable to adaptive advantages conferred by specific drug resistance mutations. CRF07_BC, on the other hand, follows a more gradual, ecologically adaptive transmission mode, implying that different epidemiological factors (e.g., population susceptibility, intensity of control measures) may differentially modulate its transmission dynamics [23]. The results indicate that since 2016, Re of CRF01_AE has fallen below 1, indicating a declining trend in its spread, consistent with the nationwide decreasing trend in CRF01_AE prevalence, likely due to effective control measures and increased public awareness [28]. In contrast, CRF07_BC, introduced to Tianjin in 2004, spread rapidly and, after a decline between 2010 and 2016, resurged, suggesting potential future spread. This may be related to diverse transmission routes, an increase in susceptible populations, and insufficient control measures [6]. Both CRF01_AE and CRF07_BC experienced rapid growth in the early 2000s, aligning with the cryptic transmission of HIV-1 in Tianjin at the time. However, CRF01_AE’s Re values showed more significant increases at different times, possibly due to its high infectivity, strong adaptability, and higher transmission efficiency in specific populations [41]. Although CRF07_BC’s Re values continue to rise, the growth is moderate, indicating that existing control measures are somewhat effective in limiting its explosive spread [20]. Nevertheless, its annual Re increase still signals enhanced transmission, necessitating strengthened monitoring and control. In conclusion, these findings emphasize the need to consider the characteristics and transmission patterns of different HIV-1 subtypes when formulating public health strategies, implementing differentiated intervention measures to effectively control and reduce HIV transmission [42]. For CRF01_AE, it is crucial to consolidate control results to prevent resurgence; for CRF07_BC, monitoring and intervention need to be intensified, especially to curb its spread among high-risk behavior groups and within transmission networks.

However, this study has certain limitations. Firstly, the research data is limited to the Tianjin area and may not fully represent the nationwide HIV-1 epidemic. Secondly, we only analyzed sequences from the pol gene region, not fully revealing the whole-genome characteristics and recombination. Future research should expand the sample scope and incorporate whole-genome sequencing technology to gain a more comprehensive understanding of HIV-1 transmission and evolution. The limitation of this study suggests that further research is needed to elucidate the specific factors contributing to the observed differences in the epidemic trajectories of CRF01_AE and CRF07_BC, and to inform evidence-based strategies for HIV-1 prevention and control.

## Conclusion

This study underscores the need for differentiated HIV prevention and control strategies tailored to the unique characteristics of the CRF01_AE and CRF07_BC subtypes. For CRF01_AE, early detection and drug resistance monitoring are critical, with an emphasis on adjusting treatment regimens promptly to mitigate the spread of drug-resistant strains. In high-prevalence areas, transitioning to integrase inhibitor-based regimens may help reduce NNRTI cross-resistance. In contrast, CRF07_BC requires focused interventions targeting young MSM groups, including sexual health education, promotion of safe sex practices, and Pre-Exposure Prophylaxis (PrEP) delivery, leveraging its concentrated transmission within specific networks. Molecular network analysis can help identify key transmission nodes for targeted interventions. Furthermore, integrating phylodynamic mapping with real-time cluster detection will enable preemptive identification of emerging hotspots, especially in provincial border regions. These findings highlight the importance of subtype-specific strategies and continuous surveillance to effectively manage HIV transmission and drug resistance.

## Supplementary Information

Supplementary material 1.

## Data Availability

The datasets used and/or analyzed during the current study are available from the corresponding author on reasonable request.

## Abbreviations

MSM: Men who have sex with men
HST: Heterosexual transmission
IDU: Injection drug users
ART: Antiretroviral therapy
AIDS: Acquired immunodeficiency syndrome
PrEP: Pre-exposure prophylaxis
SDRM: Surveillance drug resistance mutation
NRTIs: Nucleoside reverse transcriptase inhibitors
NNRTIs: Non-nucleoside reverse transcriptase inhibitors
PIs: Protease inhibitors
CRFs: Circulating recombinant forms
Re: Effective reproductive number
BDSKY: Birth-death skyline model
MCC: Maximum clade credibility
CRFs: Circulating recombinant forms
MCMC: Markov chain monte carlo
95%HPD: 95% Highest probability density
tMRCA: Time to most recent common ancestor
RPV: Rilpivirine
EFV: Efavirenz
NFV: Nelfinavir
LPV/r: Lopinavir/ritonavir
