# Supplementary material for: Divergent transmission dynamics and drug resistance evolution of HIV-1 CRF01_AE and CRF07_BC in Tianjin, China (2013–2022)
Source: Virol J. 2025 May 8;22:137. doi: 10.1186/s12985-025-02704-y (PMC12063264; doi:10.1186/s12985-025-02704-y)
Supplement: Supplementary file 1 — Supplementary material 1. [file 12985_2025_2704_MOESM1_ESM.docx]

**supplementary materials**

**Table Supplementary 1:** Factors of associated with clustering

| **Category** | **All clustered sequences** | | | **χ2** | ***P* Value** |
| --- | --- | --- | --- | --- | --- |
|  | **The whole**  **sequences**  **No. (%)** | **CRF01_AE**  **No. (%)** | **CRF07_BC**  **No. (%)** |  |  |
| **Total** | 552(100%) | 256(46.38%) | 296(53.62%) |  |  |
| **Age** |  |  |  | **8.228** | **0.039*** |
| <30 | 233(42.21%) | 97(37.89%) | 136(45.95%) |  |  |
| 30~ | 207(37.5%) | 94(36.72%) | 113(38.18%) |  |  |
| 45~ | 91(16.49%) | 53(20.7%) | 38(12.84%) |  |  |
| 60~ | 21(3.8%) | 12(4.69%) | 9(3.04%) |  |  |
| **Gender** |  |  |  | 1.539 | 0.464 |
| Famale | 1(0.18%) | 1(0.39%) | 0(0%) |  |  |
| Male | 551(99.82%) | 255(99.61%) | 296(100%) |  |  |
| **Transient population** |  |  |  | 2.524 | 0.114 |
| No | 345(62.5%) | 169(66.02%) | 176(59.46%) |  |  |
| Yes | 207(37.5%) | 87(33.98%) | 120(40.54%) |  |  |
| **Marrital status** |  |  |  | 3.201 | 0.205 |
| Single | 355(64.31%) | 159(62.11%) | 196(66.22%) |  |  |
| Married | 81(14.67%) | 45(17.58%) | 36(12.16%) |  |  |
| Divorced/widowed | 116(21.01%) | 52(20.31%) | 64(21.62%) |  |  |
| **Risk group** |  |  |  | 1.539 | 0.464 |
| HST | 9(1.63%) | 5(1.95%) | 4(1.35%) |  |  |
| MSM | 541(98.01%) | 251(98.05%) | 290(97.97%) |  |  |
| MSM+HST | 2(0.36%) | 0(0%) | 2(0.68%) |  |  |
| IDU | 0(0%) | 0(0%) | 0(0%) |  |  |
| **Education** |  |  |  |  |  |
| Junior middle school and below | 153(27.72%) | 74(28.91%) | 79(26.69%) |  |  |
| High or technical school | 147(26.63%) | 72(28.13%) | 75(25.34%) |  |  |
| Junior college or above | 252(45.65%) | 110(42.97%) | 142(47.97%) |  |  |
| **STDs** |  |  |  | 1.735 | 0.412 |
| With | 13(2.36%) | 8(3.13%) | 5(1.69%) |  |  |
| Without | 414(75%) | 194(75.78%) | 220(74.32%) |  |  |
| Unkown | 125(22.64%) | 54(21.09%) | 71(23.99%) |  |  |
| **First CD4 count (10^9^/L)** |  |  |  | 3.375 | 0.073 |
| <350 | 296(53.62%) | 148(57.81%) | 148(50%) |  |  |
| >=350 | 256(46.38%) | 108(42.19%) | 148(50%) |  |  |
| **SDRMs** |  |  |  | **7.506** | **0.009*** |
| With | 23(4.17%) | 17(6.64%) | 6(2.03%) |  |  |
| Without | 529(95.83%) | 239(93.36%) | 290(97.97%) |  |  |
